# Effectiveness of Using Gum Arabic for Co-Microencapsulation of *Ruellia tuberosa* L. and *Tithonia diversifolia* Extracts as Encapsulating Agent and Release Studies

**DOI:** 10.1155/2024/9097238

**Published:** 2024-05-24

**Authors:** Nabila Almayda, Masruri Masruri, Anna Safitri

**Affiliations:** ^1^Department of Chemistry, Faculty of Mathematic and Natural Sciences, Brawijaya University, Jl. Veteran, Malang 65145, Indonesia; ^2^Research Center for Smart Molecules of Natural Genetic Resources (SMONAGENES), Brawijaya University, Jl. Veteran, Malang 65145, Indonesia

## Abstract

This study used a combination of leaves extracts from *Ruellia tuberosa* L. and *Tithonia diversifolia* plants encapsulated using gum Arabic. The selection of leaves in medicinal plants because they are rich in bioactive compounds that provide health benefits. The encapsulation technique was microencapsulation through freeze-drying, since the nanoencapsulation for the plant extracts is unlikely to be conducted due to their large particle sizes. The resulting microcapsules were then tested their biological activities *in vitro*. Several conditions affect microcapsules' production, including pH, gum Arabic concentration, and stirring time were assessed. The optimum conditions were chosen based on the highest encapsulation efficiency. The results showed that the optimum microcapsules preparation was achived at pH 5, gum Arabic concentration of 4% (w/v), and stirring time of 60 min with an encapsulation efficiency of 84.29%. The *in vitro* assays include inhibition of alpha-amylase and antioxidant activities, resulted in the respective IC_50_ values of 54.74 *μ*g/mL and 152.74 *μ*g/mL. Releases of bioactive compounds from the microcapsules were investigated under pH 2.2 and pH 7.4 from 30 to 120 min. Results indicated a release of 43.10% at pH 2.2 and 42.26% at pH 7.4 during 120 min, demonstrating the controlled release behavior of the encapsulated bioactive compounds; nonetheless, their release behavior was not pH-dependent. This study confirms that microencapsulation has an important role in the development of plant extracts with maintained biological functions as well as maintaining their stability.

## 1. Introduction

Diabetes mellitus is a metabolic disorder characterized by the body's inability to produce or respond to insulin properly, so it cannot regulate blood sugar levels at normal levels [1]. Based on data from the International Diabetes Federation (IDF), the prevalence of type 2 diabetes mellitus (T2DM) jumped from 7.3 million in 2009 to 10.3 million in 2017 [2]. In Indonesia, T2DM is the sixth highest cause of death. About 90% of global diabetes mellitus cases fall into the category of T2DM. The number of people with T2DM continues to increase, especially among adults aged over 30 years with various social and economic backgrounds [3]. Type 2 diabetes mellitus can occur due to damaged insulin secretion by pancreatic beta cells and the inability of insulin-sensitive tissues to respond to insulin. Treatment strategies involve inhibition of alpha-amylase and increased antioxidant activity [4].

Alpha-amylase is a targeted enzyme in the treatment of T2DM [5]. Alpha-amylase involves catalysing the alpha-(1,4)-D-glycosidic linkage present in starch to hydrolyse it into smaller fragments and other glucose polymers. Inhibition of alpha-amylase activity can lower glucose levels in the blood [6]. Hyperglycemia that often occurs in people with T2DM can increase the production of free radicals in cells, causing oxidative stress. Therefore, antioxidants are needed to combat oxidative stress, thus preventing and repairing free radical damage [7]. Treatment of diabetes mellitus generally involves the use of conventional medications, such as alpha-glucosidase inhibitors, biguanides, sulfonylureas, and receptor-*γ* agonists activated by peroxisome proliferators (PPAR*γ*). However, the use of such drugs poses various problems, such as severe hypoglycemia, weight gain, and low therapeutic efficacy [8].

Some previous studies proposed that *Ruellia tuberosa* L. and *Tithonia diversifolia* as medicinal plants that have antioxidant content and have been known as antidiabetic drugs [9, 10]. *Ruellia tuberosa* L. contains large amounts of phytochemicals, such as phenols, alkaloids, flavonoids, steroids, and terpenoids [11]. Flavonoid compounds in *R. tuberosa* L. such as cirsimaritin, cirsimarin, cirsiliol 4-glucoside, sorbifolin, and pedalitin show antioxidant activity [12]. *T. diversifolia* contains active compounds such as flavonoids and sesquiterpenes lactone [13]. There are flavonoids in *T. diversifolia* that function as antioxidants such as 3,7-dimethylquercetin, retuscin, pachypodol, and chrysosplenetin [14]. The use of plant extracts as raw materials for medicines is increasing but poses several challenges such as the physical and chemical stability of extracts, as well as the efficiency of absorption by the body. Along with the times, there is microencapsulation technology that serves to increase the stability and effectiveness of compounds in drug delivery [15].

This study combined two leaves extracts from *R. tuberosa* L. and *T. diversifolia* plants with gum Arabic polymers through a microencapsulation process. The merger of these two plants has never been done, and thus, it is expected that the active compound content of both plants can produce microcapsules that have optimal benefits. Microencapsulation by techniques such as freeze-drying can protect bioactive compounds from damage and improve stability [16, 17]. Gum Arabic as a natural polymer was chosen for its low viscosity, emulsion forming ability, environmental friendliness, and nontoxicity [18]. The microencapsulation process is influenced by various factors, such as temperature, pH, crosslinking compound concentration, stirring time, stirring speed, and polymer concentration [19]. Optimization of microcapsules can be done by finding the best formulation of related parameters. The formulation of microcapsule parameters with the highest encapsulation efficiency value is then referred to as the optimum microcapsule condition [20].

This study aims to investigate the encapsulation process using freeze-drying technique. The coating material used is gum Arabic polymer. Several factors such as pH, gum Arabic concentration, and stirring time were investigated to determine optimal microencapsulation conditions. Furthermore, to determine its potential as a candidate for DMT2 drugs, biological activity tests were performed on optimal condition microcapsules through alpha-amylase activity inhibition test and antioxidant test. Drug delivery testing is also carried out with a microcapsule active ingredient release test. Characterization using Scanning Electron Microscope (SEM), Particle Size Analyzer (PSA), and Fourier Transform Infrared (FTIR) was carried out to determine the morphology, size, and identification of microcapsule functional groups.

## 2. Materials and Methods

### 2.1. Chemical and Reagents

The materials employed in the research were purchased from Merck, include gum Arabic (from the acacia tree, branched polysaccharide), methanol (ACS), acarbose (95%), alpha-amylase from *Aspergillus oryzae* (150 units/mg protein), glacial acetic acid (pharmaceutical primary standard), D-(+) glucose (analytical standard), 3,5-dinitrosalicylic acid (DNS) reagent (98%, HPLC grade), sodium hydroxide (98%, pellets, anhydrous), sodium acetate (anhydrous, 99%), sodium sulfite (98%), potassium sodium tartrate tetrahydrate (ACS reagent, 99%), quercetin (95% HPLC, solid), ascorbic acid (pharmaceutical secondary standard), sodium acetate (anhydrous, 99%), 2,2-diphenyl-1-picrylhydrazyl (DPPH), citric acid (ACS reagent, ≥99.5%), sodium citrate (ACS reagent, ≥99.0%), and aluminium chloride (anhydrous, powder, 99.99% trace metals basis). The *R. tuberosa* L. and *T. diversifolia* leaves were collected by UPT Materia Medica Batu, East Java, Indonesia with a species determination letter.

### 2.2. Instrumentation

Some of the instrumentation used in this study was Scanning Electron Microscope (SEM with TM 3000 Hitachi), CILAS 1090 Particle Size Analyzer (PSA), and Fourier Transform Infrared Spectrometer (FTIR with type Shimadzu Prestige 21).

### 2.3. Extract Preparation

Leaves powder of *R. tuberosa* L. and *T. diversifolia* was derived from the Herbal Laboratory of Materia Medica, East Java, Indonesia. A 100 g of leaves powder of *R. tuberosa* L. underwent maceration with aqueous solution at a ratio of 1 : 4 for 24 h. Simultaneously, 100 g of *T. diversifolia* leaves were macerated using an aqueous solution at a ratio of 1 : 10 for the same duration. The resulting extracts were filtered using a hydraulic press and filter paper. Subsequently, the water extracts were concentrated using a rotary evaporator operating at a speed of 70 rpm and a temperature of 90°C [13]. For further analysis, 1 g of each *R. tuberosa* L. and *T. diversifolia* extracts were dissolved in 100 mL of aqueous solution and stirred at 500 rpm. The resulting extract was stored at 4°C for further analysis.

### 2.4. Microencapsulation Procedures

The combined extract (0.1 g) was dissolved with 17.5 mL of distilled water. Subsequently, 50 mL of 4% gum Arabic solution (w/v) in buffer solution with varying pH (3, 4, 5, and 6) was gradually and stirred at 500 rpm using a magnetic stirrer. Following this, 182.5 mL of distilled water was added and stirred again with a magnetic stirrer for 60 min. The resulting microcapsules underwent freeze-drying. This procedure was replicated with different concentrations of gum Arabic solution at 2%, 4%, 6%, and 8% (w/v). The initial pH that contributing to optimal encapsulation efficiency is used, while other conditions remained consistent. Additionally, the effect of stirring time was investigated by repeating the procedure with durations of 30, 60, 90, and 120 min. The concentration of gum Arabic remained constant at 4% (w/v), a value chosen based on findings from a prior study. Optimum conditions were determined based on the percentage of encapsulation efficiency, as shown in the following equation:

(1) $$\begin{array}{c} \text{Encapsulation Efficiency}=\frac{\text{Total flavonoid content in microcapsules}}{\text{Total flavonoid content in extracts}}\;x\;100\%. \end{array}$$

### 2.5. Determination of Total Flavonoid Levels

Determination of total flavonoid levels was carried out using the calorimetry method [21]. Quercetin was utilized to generate standard calibration curves for the quantification of flavonoids. The stock solution of the combination extract and microcapsules were prepared by dissolving into 3 mL of distilled water weighing 2.5 mg extract and 5 mg microcapsules, respectively. The solution was incubated for 45 min at 40°C, then centrifuged for 2 min. The supernatant (0.6 mL) was added with 0.6 mL of 2% aluminium chloride and the mixture was incubated for 23 min at room temperature. The wavelength used to measure the absorbance of the solution is 420 nm which was obtained from the maximum wavelength of quercetin. Total flavonoid levels were determined using a quercetin standard curve plot (mg QE/g).

### 2.6. Alpha-Amylase Enzyme Inhibition Test

Samples (acarbose, extracts, and microcapsules) were prepared in various concentrations of 20, 40, 60, 80, and 100 *μ*g/mL. 250 *μ*L of each concentration was taken and put into a reaction tube. Alpha-amylase enzyme (50 *μ*g/mL) as much as 250 *μ*g/mL was added to the sample and incubated for 30 min at 37°C. 250 *μ*L of 1% (w/v) starch solution was added and incubated again for 10 min at 25°C. DNS reagent (500 *μ*L) was added to the solution. The solution was incubated in boiling water until a brownish red colour formed (±5 min), and the solution was cooled with the addition of 5 mL of distilled water. The results of determining the maximum wavelength of reduced DNS (480 nm) were used to measure the absorbance of the solution. The percentage of alpha-amylase enzyme inhibition was calculated using the following formula:

(2) $$\begin{array}{c} \text{Percentage of Enzyme Inhibition}=\frac{\text{Absorbance control}-\text{Absorbance sample}}{\text{Absorbance control}}\;x\;100\%. \end{array}$$

The sample concentration (*x*) and percent inhibition (*y*) are plotted on a curve to create a linear regression equation, so that the IC_50_ value of the sample can be known. The IC_50_ value for each sample corresponds to the concentration (*x*) at which enzyme inhibition is 50%, expressed mathematically as the intersection point of the regression equation with *y* = 50.

### 2.7. Antioxidant Activity Test

Different concentrations of ascorbic acid (1, 4, 7, 10, and 13 *μ*g/mL), extract samples (20, 40, 60, 80, and 100 *μ*g/mL), microcapsules (120, 140, 160, 180, and 200 *μ*g/mL) were prepared. A 3 mL solution from each sample concentration was transferred into a dark vial. Subsequently, 2 mL of DPPH (2,2-diphenyl-1-picrylhydrazyl) solution was added. For the control solution, 3 mL of ethanol was taken, placed into a dark vial, and then mixed with 2 mL of DPPH solution. The mixtures were homogenized and left to incubate for 20 min in a dark environment, free from light to minimize factors that could influence the DPPH method. The maximum wavelength of DPPH (516 nm) was used to measure the absorbance of the sample. The percentage of sample antioxidants was determined from the following equation:

(3) $$\begin{array}{c} \text{Percentage of Antioxidant}=\frac{\text{Absorbance control}-\text{Absorbance sample}}{\text{Absorbance control}}\;x\;100\%. \end{array}$$

Determination of IC_50_ values for antioxidant activity followed a similar approach to the alpha-amylase inhibition assay. The sample concentration is plotted on the *x*-axis, and the percentage of antioxidant activity was plotted on the *y*-axis. The linear regression equation obtained from the curve is then used to determine the IC_50_ value.

### 2.8. *In Vitro* Release Study

The medium used in the test for the release of the active ingredient in the microcapsule combination of *R. tuberosa* L. and *T. diversifolia* extracts was phosphate buffer saline. The PBS used was pH 2.2 and pH 7.4. PBS (10 mL) was prepared and conditioned at a temperature of 37°C. Then, sample (0.1 g) was added and stirred constantly at a speed of 100 rpm. At time intervals of 30-, 60-, 90-, and 120-min samples (2.5 mL) were taken, and their absorbance was measured using the maximum wavelength of quercetin (420 nm). The release from the sample was calculated using the following equation:

(4) $$\begin{array}{c} \text{Percentage of Release}=\frac{\text{Total flavonoid content release from microcapsules}}{\text{Total flavonoid content in microcapsules}}\;x\;100\%. \end{array}$$

### 2.9. Characterization of Microcapsules

Characterization of microcapsules was carried out using SEM, FTIR, and PSA instruments. Hitachi TM 3000 Scanning Electron Microscope was used to determine the morphology of the microcapsules before and after extracts released from the microcapsules, with magnifications ranging from 500 to 15,000×. Fourier Transform Infrared (FTIR) Shimadzu-Type IR Prestige-21 was used to determine functional groups in gum Arabic, extracts and microcapsules by first drying the samples and compressing them into KBR pellets, then measuring them in the wave number range of 4,000–400 cm^−1^. CILAS 1090 Particle Size Analyzer was used to determine the size of microcapsules.

### 2.10. Data Analysis

Statistical analysis was carried out using Statistical Package for the Social Sciences (SPSS) v.26 software. The data was statistically measured and described through narratives and tables. Microencapsulation outcomes involving variations in pH, coating concentration, and stirring time underwent analysis using the Kolmogorov-Smirnov test for normality. Furthermore, the data from the test results was subjected to one-way analysis of variance (ANOVA) with a confidence level of 95% (*α* = 0.05). These statistical tests were performed under the assumptions of normal data distribution and homogeneous data variation.

## 3. Results

### 3.1. Microencapsulation of Combination of *R. tuberosa* L. and *T. diversifolia* Extracts

Microencapsulated extracts of a combination of *R. tuberosa* L. and *T. diversifolia* were produced by freeze-drying method.  Table 1 provides details on the encapsulation efficiency of microcapsules produced under varying pH levels, gum Arabic concentrations, and stirring times. Notably, microcapsules containing a combination extract of *R. tuberosa* L. and *Tithonia diversifolia*, prepared at pH 5, with a gum Arabic concentration of 4% (w/v), and a stirring time of 60 min, exhibited the highest percentage of encapsulation efficiency. Microcapsules produced under these specific conditions undergo further analysis.

### 3.2. Characterization of Microcapsules

**Figure 1:**
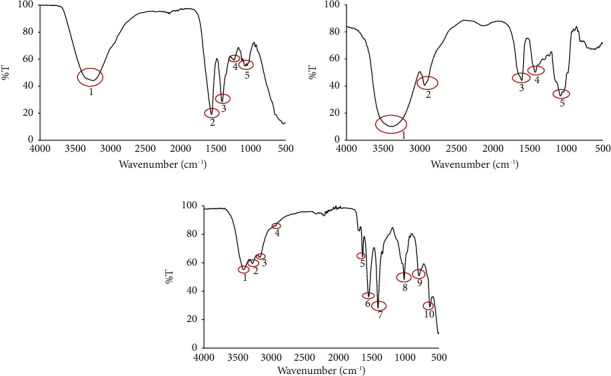
FTIR spectra of the (a) combination of *R. tuberosa* L. and *T. diversifolia* extract; (b) gum Arabic; (c) microcapsules combination of *R. tuberosa* L. and *T. diversifolia* extract prepared in pH 5, 4% (w/v) gum Arabic, and 60 min stirring time.

The analysis of the microcapsules containing a combination of *R. tuberosa* L. and *T. diversifolia* extract was initially conducted through Fourier Transform Infrared (FTIR).  Figure 1 exhibits the FTIR spectra and the interpretation of the FTIR spectra is provided in Table 2.

**Figure 2:**
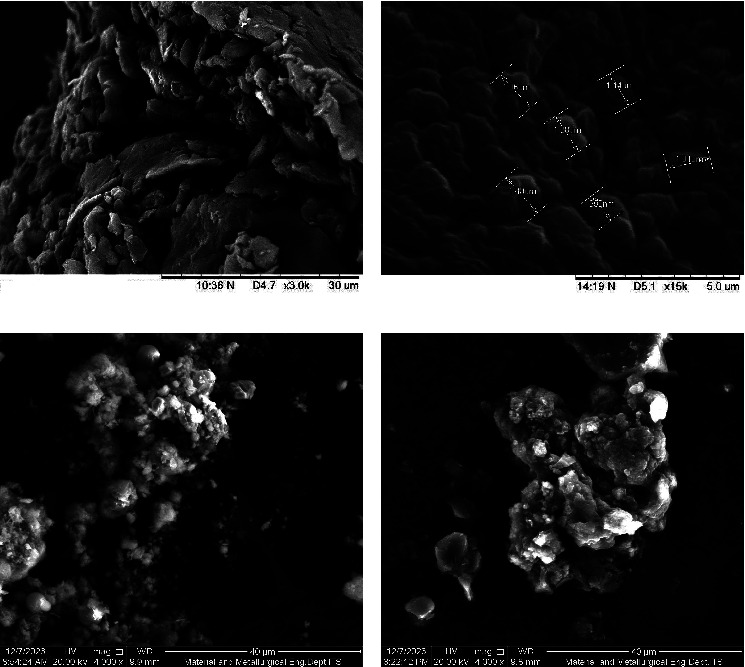
SEM images of the: (a) combination of *R. tuberosa* L. and *T. diversifolia* extracts; (b) microcapsules combination of *R. tuberosa* L. and *T. diversifolia* extract prepared in pH 5, 4% (w/v) gum Arabic, and 60 min stirring time; (c) microcapsules combination of *R. tuberosa* L. and *T. diversifolia* extract after release test at medium pH 2.2; (d) microcapsules combination of *R. tuberosa* L. and *T. diversifolia* extract after release test at medium pH 7.4.

The morphological analysis, conducted through Scanning Electron Microscopy (SEM), is presented in Figure 2. SEM was employed to characterize the morphology of both extracts and microcapsules. The images highlight the distinctions in microcapsule morphology under initial conditions, as well as microcapsules that have been released in acidic and neutral pH mediums, simulating conditions within the human body.

**Figure 3:**
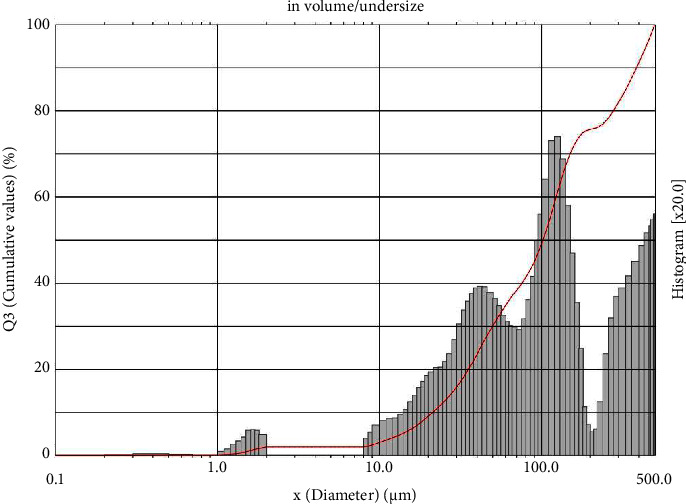
Particle size distribution from microcapsules combination of *R. tuberosa* L. and *T. diversifolia* extract prepared in pH 5, 4% (w/v) gum Arabic, and 60 min stirring time, the mean diameter was 145.88 *μ*m.

**Figure 4:**
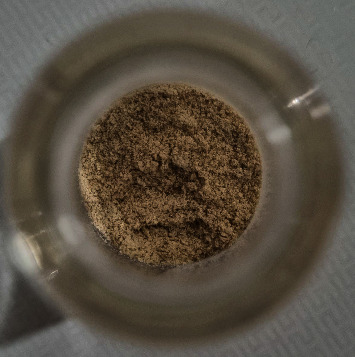
Microcapsules powder from the combination of *R. tuberosa* L. and *T. diversifolia* extract prepared in pH 5, 4% (w/v) gum Arabic, and 60 min stirring time.

The characterized microcapsules are further analysed for their size and surface structure. Particle size distribution analysis provides insights into the behavior and stability of microcapsules. The results of this analysis, conducted using PSA, are presented in Figure 3. The microcapsules produced in this research are in powder form shown in Figure 4. The freeze-drying process is used to convert liquid microcapsules into powder to make them easier to store and use in various applications.

### 3.3. Biological Activity Test of Microcapsules

**Figure 5:**
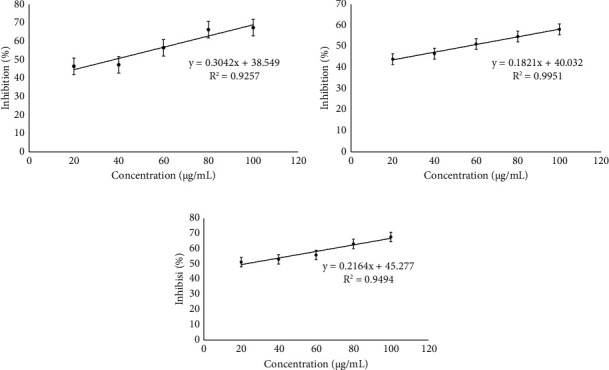
The alpha-amylase inhibition of: (a) combination of *R. tuberosa* L. and *T. diversifolia* extract, with IC_50_ 37.64 ± 0.94 *μ*g/mL; (b) microcapsules combination of *R. tuberosa* L. and *T. diversifolia* extract prepared in pH 5, 4% (w/v) gum Arabic, and 60 min stirring time, with IC_50_ 54.74 ± 5.31 *μ*g/mL; (c) acarbose, with IC_50_ 21.83 ± 3.14 *μ*g/mL.

**Figure 6:**
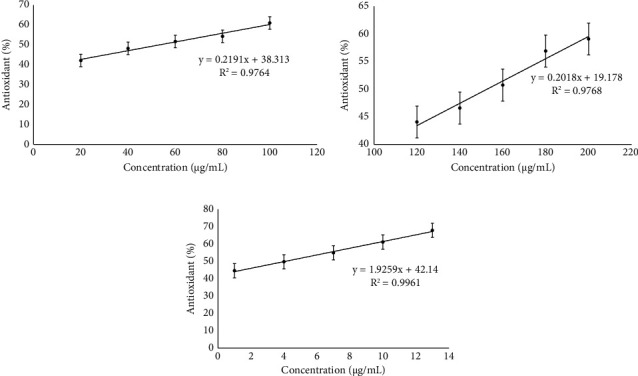
The antioxidant activity of: (a) combination of *R. tuberosa* L. and *T. diversifolia* extracts, with IC_50_ 53.34 ± 1.36 *μ*g/mL; (b) microcapsules combination of *R. tuberosa* L. and *T. diversifolia* extract prepared in pH 5, 4% (w/v) gum Arabic, and 60 min stirring time, with IC_50_ 152.74 ± 1.49 *μ*g/mL; (c) ascorbic acid, with IC_50_ 4.08 ± 0.08 *μ*g/mL.

The biological activity of the microcapsules was assessed through alpha-amylase inhibition and antioxidant analysis. Figures 5 and 6 present the outcomes of *in vitro* biological testing. Acarbose and ascorbic acid served as positive controls in the alpha-amylase inhibition assay and antioxidant assay, respectively. Additionally, the combination extracts of *R. tuberosa* L. and *T. diversifolia* (nonencapsulated) were also tested for comparison with the microcapsules.

### 3.4. Release Test of Microcapsules

**Figure 7:**
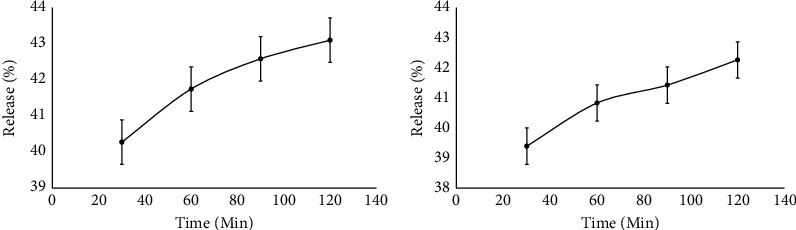
Graphs of bioactive compounds released from microcapsules combination of *R. tuberos*a L. and *T. diversifolia* extracts prepared in pH 5, 4% (w/v) gum Arabic, and 60 min stirring time: (a) pH 2.2 and (b) pH 7.4.

The *in vitro* analysis of the microcapsules included an examination of the release of active ingredients from the combined extracts of *R. tuberosa* L. and *T. diversifolia*.  Figure 7 illustrates the release profiles of microcapsules in two different mediums, specifically at pH 2.2 and pH 7.4.

## 4. Discussion

Microencapsulation combination of *R. tuberosa* L. and *T. diversifolia* extracts was carried out by freeze-drying method. The principle of freeze-drying (lyophilization) involves the removal of water from a sample by utilizing the phase change from ice to steam without passing through the liquid phase. This process consists of three main stages: freezing, sublimation, and desorption. This method has the advantage of maintaining the quality of drying results [17]. Microencapsulation allows to increase the stability of the active ingredients, control release, and allows directional delivery to the desired location in the body. This can help in increasing the effectiveness of treatment, reducing unwanted side effects, and allowing the use of therapies tailored to the needs of patients [22]. In microcapsules preparations, there are several factors that affect microencapsulation, including the pH of the medium, temperature, type and concentration of coating material, concentration of crosslinking compounds, and stirring time and speed [19].

Table 1 shows the percentage encapsulation efficiency of microcapsules made variously including pH, gum Arabic concentration, and stirring time. Optimization is carried out to determine the best conditions that lead to the formation of microcapsules with a high percentage of encapsulation efficiency. The optimum conditions obtained in making microcapsules are at pH 5, gum Arabic concentration of 4% (w/v), and stirring time of 60 min. Microcapsules with optimum conditions that will be used for further analysis related to their biological activity test.

The stability of gum Arabic as a polymer is influenced by pH due to the ionic nature of its molecules. Gum Arabic contains carboxylic acid groups and hydroxyl groups that play a role in acid-base reactions (the pKa of Arabic gum is close to 3.6). Changes in pH can affect the electrical charge on gum Arabic molecules, thus affecting intermolecular interactions and overall polymer structure. Gum Arabic, as an amphoteric polysaccharide, can ionize to carboxylic acid groups at low pH (forming a negative charge) and hydroxyl groups at high pH (forming a positive charge), thus allowing it to dissolve in water. The results of the study showed that pH variations in the microencapsulation process of the combination of *R. tuberosa* L. and *T. diversifolia* extracts can affect the encapsulation efficiency. At pH 5, the encapsulation efficiency increases because the acid groups in the gum Arabic have not been fully ionized, resulting in low viscosity and optimal interaction. The reduced efficiency is possible due to differences in the number of COOH groups in the gum Arabic molecule [23, 24].

In this study, optimal microcapsules conditions were obtained at a gum Arabic concentration of 4% (w/v). The concentration of polymer used affects the size of the microcapsule, the value of encapsulation efficiency, and the percentage of drug release. Higher concentrations of gum Arabic result in lower encapsulation efficiency, as shown in Table 1. According to Wibowo et al. [25], a lower polymer concentration will form a larger pore size, thus making it easier for the active ingredients to enter the microcapsule and will automatically also increase the percentage of encapsulation efficiency.

The optimum stirring time is determined based on the largest percent efficient encapsulation shown in Table 1. Microencapsulation performed with stirring time variations of 30, 60, 90, and 120 min showed different percent encapsulation efficiency. The optimum stirring time is obtained at 60 min. The length of stirring time will affect the size and surface area of the microcapsule formed. Microcapsules with smaller size and larger surface area will more easily coated the active ingredients into the matrix and increase the percentage of encapsulation efficiency formed [26].

Analysis of the characteristics of the microcapsule is carried out to determine the chemical structure, surface morphological structure, and particle size of the microcapsule. Characterization was carried out using FTIR (Fourier Transform Infrared) spectrophotometry to identify the constituent functional groups of microcapsules, SEM (Scanning Electron Microscope) to see the surface morphology of the microcapsule, and PSA (Particle Size Analyzer) to determine the size distribution of microcapsules.  Figure 1 shows the FTIR spectra of the microcapsule at optimum conditions compared to the spectra of the combination of extracts (*R. tuberosa* L. and *T. diversifolia*) and gum Arabic, while the functional group identification of the three samples is presented in Table 2. Based on the spectral data of the extract and gum Arabic as seen in Figure 1, there is a new peak in the microcapsule, namely, at peak 2. Peak 2 at wavenumber 2926.59 cm^−1^ indicates the presence of C-H stretching thought to be derived from aliphatic C-H in gum Arabic. If the optimum microcapsule spectra are compared with the gum Arabic spectra and extract spectra, then the optimum microcapsule spectra are more similar to the gum Arabic. This shows that the microencapsulation process carried out was successful because the extract was coated with gum Arabic [27].

In this study, the size and surface structure of microcapsules containing a combination of *R. tuberosa* L. and *T. diversifolia* extracts appeared to be nearly identical to common microcapsules. Similar findings have been observed by other researchers that produce microcapsules of small, irregular size, with extensive wrinkles, and a more toothy surface produced through the freeze-drying process [28]. The irregular shape of the surface can be caused by the low drying temperature when the microcapsules are dried by freeze-dryer. Low temperatures can form ice crystals which will then trigger the formation of cavities in the microcapsule and give rise to SEM images with irregular surface morphology [29]. However, this microcapsule product has reached the size range that generally occurs in microcapsules, with an average diameter of about 145.88 *μ*m.

The freeze-drying microencapsulation resulted in powder form of microcapsules, as shown in Figure 4. In the freeze drying, liquid microcapsules solution was converted into powder, and hence easier to apply for many applications. Microcapsules are often produced in powder form for several reasons, including providing protection from environmental factors and designing to release the active ingredient in a controlled manner as needed. This allows for slow or rapid release of the active ingredient, according to the purpose of the product [30, 31].

The biological activity test of microcapsules was carried out in vitro using alpha-amylase inhibition test and antioxidant activity test. From these tests, it is known that the combination microcapsules of *R. tuberosa* L. and *T. diversifolia* extracts still have high biological activity. Inhibition of the activity of the enzyme alpha-amylase can slow down the digestion of carbohydrates and the absorption of glucose in the body. The alpha-amylase enzyme inhibition test was performed to see the inhibitory ability produced by microcapsules made under optimum conditions. The test results are displayed and expressed in IC_50_ values in Figure 5. Microcapsules made at optimum conditions produced the highest IC_50_ value of 54.74 ± 5.31 *μ*g/mL, followed by extract and acarbose with IC_50_ values of 37.64 ± 0.94 *μ*g/mL and 21.83 ± 3.14 *μ*g/mL respectively. Based on the IC_50_ value in the table above, all three samples were able to produce inhibitory activity against the alpha-amylase enzyme. Acarbose was shown to have the strongest inhibitory activity against alpha-amylase enzymes, while microcapsule samples had the weakest inhibitory activity against similar enzymes. The combination of *R. tuberosa* L. and *T. diversifolia* extracts produced a smaller resistance than acarbose, but larger than microcapsules made under optimum conditions. From the test results, it is known that the optimum condition microcapsule sample has low activity inhibition of alpha-amylase enzyme because some bioactive compounds are still retained in the microcapsule matrix and cannot be released completely [26, 31].

Biologically active compounds, such as flavonoids, saponins, phenolic compounds, terpenes, and cardiac glycosides, provide a number of health benefits and potentially as natural antioxidants that can fight free radicals and reactive oxygen species (ROS). Antioxidant activity testing is one of the parameters that can be used to assess the antidiabetic potential of a drug candidate. This is due to the frequent link between diabetes and the presence of free radicals. Antioxidants act as compounds that can protect the body from the damaging effects of free radicals [32, 33]. The antioxidant activity test was carried out using the DPPH method (2,2-diphenyl-1-picrylhydrazyl). DPPH radicals are organic compounds that contain unstable nitrogen and produce a dark purple solution [34]. Based on the test results shown in Figure 6, the optimum condition microcapsule sample has a value IC_50_ of 152.74 ± 1.49 *μ*g/mL. The IC_50_ value of the microcapsule sample was greater than the extract combination sample of 53.34 ± 1.36 *μ*g/mL and ascorbic acid of 4.08 ± 0.08 *μ*g/mL. This indicates that the optimum condition microcapsule sample has the smallest antioxidant activity. The difference in antioxidant activity between samples of optimum condition microcapsules with the combination of extract and ascorbic acid can be influenced by the concentration of bioactive compounds contained in each sample. The more bioactive compounds contained, the stronger the antioxidant activity, while the optimal condition microcapsule sample is classified as medium antioxidant. This shows that the content of bioactive compounds contained in microcapsules is smaller, so that to be able to inhibit DPPH radical activity by 50%, a larger concentration of microcapsule samples is needed. Moderate levels of antioxidant activity may still be sufficient to provide protection against oxidative stress and help reduce the risk of various diseases associated with free radicals, including degenerative diseases and cardiovascular disease [34]. In addition, moderate antioxidant activity can also be caused by bioactive compounds that are still retained in microcapsules. Therefore, the number of bioactive compounds that can neutralize DPPH radicals is less.

The release test is carried out to see the speed of dissolution of the active ingredient in the microcapsule of the *R. tuberosa* L. extract from a solid preparation into a certain medium. This test also needs to be done to show whether microencapsulation successfully has a good delivery system in the gastrointestinal tract. Tests were conducted on PBS medium pH 2.2 and pH 7.4 with discharge times at min of 30, 60, 90, and 120. At the time of the discharge test, stirring is carried out at a speed of 100 rpm with temperature 37°C. Stirring is carried out at a speed of 100 rpm to function so that the drug released through the membrane is faster during the dissolution process. During the testing process, the temperature is maintained at 37 ± 5°C because it describes the temperature of the human body [19–21]. Phosphate buffer saline (PBS) is commonly used in release tests because it mimics the physiological condition of the human body [35].  Figure 7 shows the release profile of microcapsules under optimum conditions at pH 2.2 and pH 7.4, while Figures 2(c) and 2(d) are the results of SEM analysis after the discharge test. At pH 2.2, a percent release of the percentage is produced by 40.27% (30 min), 41.75% (60 min), 42.58% (90 min), and 43.10% (120 min), while at pH 7.4, the percent release is produced by 39.39% (30 min), 40.83% (60 min), 41.43% (90 min), and 42.26% (120 min). Based on the statistical analysis, this shows that pH has no effect on the release process of microcapsule active ingredients.

The polymer used affects the process of releasing drugs from microparticles. Gum Arabic is an amphiphilic polysaccharide. Gum Arabic is widely used in many applications because it exhibits high solubility and low viscosity at high concentrations and has good mulching properties, as well as microencapsulation. Arabic gum contains carboxylate groups on its molecules. The carboxylate group has the ability to release a proton (H^+^), producing a negatively charged carboxylate ion (COO^−^). When the pH is above 2.2, the carboxylate group in the gum Arabic can undergo dissociation of the carboxyl group in its molecular chain. However, some studies have shown that gum Arabic lacks long-term stability against oxidation [36, 37]. According to research conducted by Ibrahim et al. [38], the discharge test showed a higher discharge in the buffer solution with a pH of 1.2 compared to a pH of 7.4., this was due to the relaxation of the polymer caused by the protonation of the acid. Based on previous research, the release of active ingredients from microcapsules is greater at pH 7.4 (blood) compared to pH 2.2 (stomach). This can be influenced by the type of polymer used in the microencapsulation process. Therefore, further research needs to be carried out regarding the combination of gum Arabic with other types of polymers; therefore, the microcapsule results obtained are more optimal [19–21].

## 5. Conclusion

The microencapsulated combination of *R. tuberosa* L. and *T. diversifolia* extracts was successfully processed using the freeze-drying technique with gum Arabic as a coating material. Optimum conditions for microcapsules were obtained at a pH of 5.4% (w/v) gum Arabic and a stirring time of 60 min. The FTIR analysis showed that there was a new peak uptake in the microcapsule under optimum conditions due to the influence of gum Arabic. Morphological analysis using SEM showed that the surface of the microcapsules was irregular due to the influence of freeze-drying, but PSA showed microsized particles with an average diameter of 145.88 *μ*m. The combination of *R. tuberosa* L. and *T. diversifolia* extract microcapsules still maintained its biological activity as inhibitors for alpha-amylase and antioxidants, with IC_50_ values of 54.74 *μ*g/mL and 152.74 *μ*g/mL, respectively. *In vitro* release analysis showed that microcapsules were released earlier at pH 2.2 than at pH 7.4. This research highlights the importance of microencapsulation in preserving the high biological activity of plant extracts during production. Nevertheless, the current study employed only one type of polysaccharide polymer. It is notable that in the future, a combination of protein and polysaccharide polymers can be used for achieving high encapsulation efficiency and high biological activity.

## Figures and Tables

**Table 1:** Encapsulation efficiency of microcapsules combination of *R. tuberosa* L. and *T. diversifolia* prepared in various pH, gum Arabic concentration, and stirring time.

Sample^*∗*^	% EE^*∗∗*^
Microcapsules prepared in pH 3	28.91 ± 1.33^a^
Microcapsules prepared in pH 4	34.78 ± 6.76^b^
Microcapsules prepared in pH 5	84.14 ± 4.19^c^
Microcapsules prepared in pH 6	19.00 ± 2.11^a^
Microcapsules prepared in gum Arabic 2% (w/v)	53.70 ± 8.98^a^
Microcapsules prepared in gum Arabic 4% (w/v)	84.29 ± 9.84^b^
Microcapsules prepared in gum Arabic 6% (w/v)	53.30 ± 3.29^a^
Microcapsules prepared in gum Arabic 8% (w/v)	45.73 ± 8.52^a^
Microcapsules prepared in 30 min stirring time	50.89 ± 10.09^a^
Microcapsules prepared in 60 min stirring time	74.32 ± 1.84^b^
Microcapsules prepared in 90 min stirring time	47.42 ± 3.92^a^
Microcapsules prepared in 120 min stirring time	41.95 ± 9.37^a^

^*∗*^Samples with variations in pH were prepared with a gum Arabic concentration of 4% and a stirring time of 60 min. Samples with variations in gum Arabic concentration were prepared at pH 5 with a stirring time of 60 min. Samples with variations in stirring time were prepared at pH 5 with a gum Arabic concentration of 4%. ^*∗∗*^Distinct notations signify (a, b, c) significant differences between conditions, as determined by the one-way ANOVA test with a confidence level of ∝ = 0.05.

**Table 2:** Interpretation of the FTIR spectra.

Peak number	Combination of *R*. *tuberosa* L. and *T. diversifolia* extract	Gum Arabic	Microcapsules combination of *R. tuberosa* L. and *T. diversifolia* extract
1	3243.21 cm^−1^ for O-H alcohol	3274.58 cm^−1^ for O-H alcohol	3408.65 cm^−1^ for O-H alcohol
2	1556.00 cm^−1^ for C=C aromatic	2926.59 cm^−1^ for C-H alkane	3278.86 cm^−1^ for O-H alcohol
3	1404.82 cm^−1^ for COO^−^ symmetric carboxylate	1598.78 cm^−1^ for C=C aromatic	3169.04 cm^−1^ for O-H alcohol
4	1236.53 cm^−1^ for C-O ester	1413.38 cm^−1^ for COO^−^ symmetric carboxylate	2935.14 cm^−1^ for C-H alkane
5	1075.36 cm^−1^ for C-O ether	1019.74 cm^−1^ for C-O ether	1637.29 cm^−1^ for C=O carbonyl
6			1546.01 cm^−1^ for N-H amide
7			1406.24 cm^−1^ for COO^−^ symmetric carboxylate
8			1016.89 cm^−1^ for C-Cl alkyl halides
9			797.25 cm^−1^ for C-H alkyl halides
10			636.09 cm^−1^ for C-Cl alkyl halides

## Data Availability

The data used to support this study are included in the article and are available from the corresponding author upon request.
